# Comparison of Shear Wave Elastography and Dynamometer Test in Muscle Tissue Characterization for Potential Medical and Sport Application

**DOI:** 10.3389/pore.2021.1609798

**Published:** 2021-06-29

**Authors:** Peter Soldos, Zsuzsanna Besenyi, Katalin Hideghéty, László Pávics, Ádám Hegedűs, Levente Rácz, Bence Kopper

**Affiliations:** ^1^ Faculty of Kinesiology, University of Physical Education, Budapest, Hungary; ^2^ Department of Nuclear Medicine, Faculty of Medicine, University of Szeged, Szeged, Hungary; ^3^ Department of Oncotherapy, Faculty of Medicine, University of Szeged, Szeged, Hungary

**Keywords:** dynamometry, muscle thickness, muscle stiffness, shear wave elastography, strength parameters

## Abstract

Skeletal muscle status and its dynamic follow up are of particular importance in the management of several diseases where weight and muscle mass loss and, consequently, immobilization occurs, as in cancer and its treatment, as well as in neurodegenerative disorders. But immobilization is not the direct result of body and muscle mass loss, but rather the loss of the maximal tension capabilities of the skeletal muscle. Therefore, the development of a non-invasive and real-time method which can measure muscle tension capabilities in immobile patients is highly anticipated. Our aim was to introduce and evaluate a special ultrasound measurement technique to estimate a maximal muscle tension characteristic which can be used in medicine and also in sports diagnostics. Therefore, we determined the relationship between the results of shear wave elastography measurements and the dynamometric data of individuals. The measurements were concluded on the m. vastus lateralis. Twelve healthy elite athletes took part in our preliminary proof of principle study—five endurance (S) and seven strength (F) athletes showing unambiguously different muscle composition features, nine healthy subjects (H) without prior sports background, and four cancer patients in treatment for a stage 3 brain tumor (T). Results showed a high correlation between the maximal dynamometric isometric torque (Mmax) and mean elasticity value (*E*) for the non-athletes [(H + T), (*r* = 0.795)] and for the athletes [(S + F), (*r* = 0.79)]. For the athletes (S + F), the rate of tension development at contraction (RTDk) and *E* correlation was also determined (*r* = 0.84, *p* < 0.05). Our measurements showed significantly greater *E* values for the strength athletes with fast muscle fiber dominance than endurance athletes with slow muscle fiber dominance (*p* < 0.05). Our findings suggest that shear wave ultrasound elastography is a promising method for estimating maximal muscle tension and, also, the human skeletal muscle fiber ratio. These results warrant further investigations with a larger number of individuals, both in medicine and in sports science.

## Introduction

Skeletal muscle status defines individual physical performance, which has great importance in healthy people and athletes, but also plays a significant role as well in terms of injuries, aging, and the acquisition of various diseases. Cancer affects patients’ muscle mass in several ways. Patients with a malignant disease frequently suffer from disease-related malnutrition and sarcopenia, brain tumors can cause locomotor damage, and some treatment modalities, such as surgery and radiotherapy, can influence the muscles directly or their innervation, leading to functional decline. Additionally, a loss of strength and muscle activity have a negative effect on treatment and clinical results, and also result in patient immobilization and hospitalization (1). An objective, non-invasive method for the assessment of the skeletal muscle compartment in cancer patients and, based on these results, the execution of specialized rehabilitation, could have a positive impact on clinical outcomes (2). The monitoring of morphological, structural, and functional changes in skeletal muscles is fundamental in oncology, neurology, geriatrics, and many other disciplines.

Determining the properties of muscle tension at maximum contraction of the human skeletal muscle in professional sports and in the closely related area of rehabilitation is also an important task (3). Early recognition of the physical abilities of young athletes is also increasingly important for the selection of the appropriate sport for a given individual. One of the most important aspects of this process is the application of modern diagnostic methods, which play an important role not only in the early screening of talent, but also in the prevention and rehabilitation of various types of injuries that may be the result of poor sport selection (4). Muscle mass change is a common phenomenon due to intensive exercise, load, or recovery process of different kind of injuries. In such cases real-time physical parameters of the muscle, its hypertrophy capability, and its maximum exertion all provide feedback to experts on the methodology employed (5–7).

Muscle effort is most often measured indirectly by using dynamometric devices in angled joint position (such as Biodex Dynamometer System 4), but in these cases the torque applied by the muscle to a given joint or limb is being determined (8–13). With knowledge of the muscle’s leverage to the joint and the measured torque, the muscular effort can theoretically be calculated. Here the situation is more complex, because in most cases several muscles simultaneously create torque in a joint, so based on the measured torque, one must take into account that the determination of the effort of a given muscle comes with a high level of uncertainty. In some cases, however, a patient’s health status or the injury of the test subject does not even allow the use of a dynamometer, even though knowledge of the muscular effort would be of a great importance. In addition, for example, after the rupture of an anterior cruciate ligament (ACL) or meniscus, the dynamometric measurement of the quadriceps femoris with a bent knee creates such a load and shear force in the knee joint which could result in the reoccurrence of the injury (14–16). With that in mind, a new technology that can measure individual muscle tension can be beneficial both in the area of clinical patient treatment and in the field of professional sports rehabilitation.

Ultrasonography is an effective diagnostic modality for the musculoskeletal system due to its ability to perform real-time dynamic high-resolution examinations. In addition to being able to acquire morphologic data, we can now obtain biomechanical information by quantifying the elasticity of the musculoskeletal structures with ultrasound (US) elastography (17).

The basic principle of shear wave elastography is to create a shear wave through a stress by acoustic radiation force, to map the tissue distortion impulse in the tissue using sonography and to trace the wave back to the mechanical properties of the tissue by using special algorithms. Stiffness is displayed on a B-mode scan with an overlaid elastogram in color and the stiffness value is measured within a region of interest (ROI) on the elastogram (18).

Shear wave elastography (SWE) is considered to be the most suitable type of US elastography for the measurement of musculoskeletal system characteristics. Elastography provides the opportunity to further deepen our understanding of the interaction between muscle structure and muscle tension (19).

Therefore, the aim of our study was to investigate the possible applications of quantitative shear wave elastography in the determination of the mechanical properties, such as maximal tension, of the skeletal muscles.

## Materials and Methods

### Subjects and Study Protocol

**FIGURE 1:**
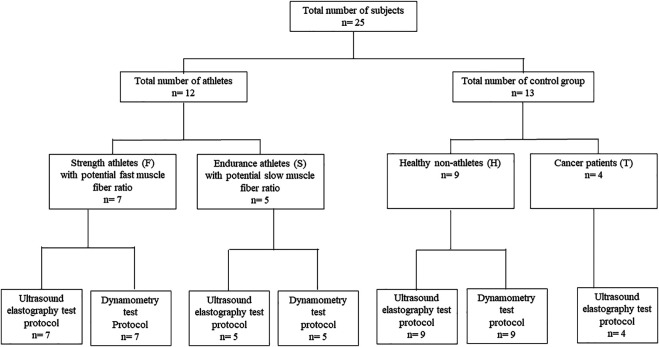
Flow diagram of research.

We conducted a preliminary investigation on January 17, 2019 with the participation of twelve top-level elite athletes (four females and eight males) with a statistical age (SA) of 22.6 ± 8.3. Five of them can be characterized as extreme endurance athletes with a high certainty of slow muscle fiber dominance (S), as this fiber composition was observed as a key element in their achieving top results in that sport (20), and seven can be characterized as strength athletes with a high certainty of fast muscle fiber dominance (F). The subjects were in good health and had no prior injuries in the muscles tested. Exclusion criteria included a known history of trauma regarding any affected extremity, a known history of previous surgery and neurological disease, and lower back pain. The reason we selected professional athletes for our study was, firstly, that as this is a preliminary study aimed at determining the existence of a correlation between muscle tension and US elastography data; for practical reasons and for the simplicity of the procedure, we did not want to use patients at this stage but only healthy participants. Secondly, by using top-level athletes, due to the nature of their sports, predicting their fiber composition characteristics was relatively easy. After our initial investigation, to widen the range of the investigated samples, we conducted a second data measurement with thirteen non-athletes with a SA of 35.01 ± 8.09, more specifically, nine healthy individuals without a prior sports background (H) and four cancer patients in treatment with glioblastoma multiforme III–IV (T). (Figure 1). As COVID restrictions were active in the hospital where we executed the data collection, the number of patients we could include in the study was limited.

According to the test protocol, we investigated the dominant lower extremity of each individual by simultaneously using US elastography (GE Logiq E9 US system with 9 MHz linear probe– General Electric, GE Healthcare) and a computer-controlled dynamometer (Multicont II Tihanyi System 2003 Hungary). Before all other measurements, we used a body composition monitor with scale (Omron Healthcare Co. Ltd, BF500: 200712-01491F) to obtain the following basic body composition data: body height (BH); body weight (BW); body mass index (BMI); body fat percentage (BF); visceral fat percentage (VF); and body muscle percentage (BM).

We saved the results electronically with the help of the equipment used in the study and exported the data for further statistical analysis.

The trial was conducted in accordance with the Declaration of Helsinki (as revised in 2013). The study was approved by the Regional and Institutional Human Biomedical Research Ethics Committee of the University Szeged (NO.: 4449) and informed consent was obtained from all individual participants.

### Dynamometry

After a 10-min warm-up on a stationary bike and 5 min of light gymnastic exercises the subjects underwent a dynamic examination of the dominant knee extensor muscles. For the knee extensor muscles, the starting position was 90° flexion and 20° flexion in the end position. We examined the magnitude of effort with an angular velocity of 20°/s, under near isometric conditions, and we determined the angular position of maximum effort. After a rest period of 10 min, the muscle was examined under the given angular position under isometric conditions. Subjects had three attempts with small, medium, and maximum effort. Maximum effort was required to be reached in 3 s. Maximal isometric torque (Mmax) was determined for all participants, and for the athletes the rate of tension development both in contraction (RTDk) and release (RTDr) was measured (21–24). We did not measure RTD values for the non-athlete (H + T) groups because it is necessary to have prior experience concerning the data collection protocol on a dynamometer while executing rapid maximal isometric contraction to minimize the risk of injury. Between the tests, 5 min of breaks were inserted to prevent fatigue on the part of the subjects from affecting the measurements.

### Imaging

**FIGURE 2:**
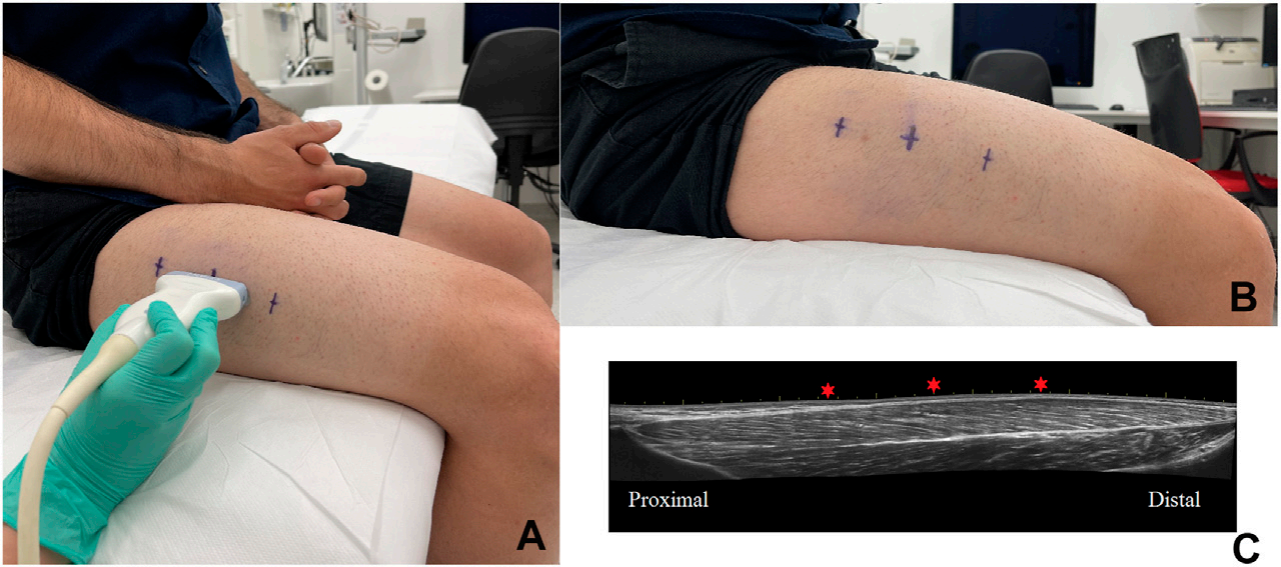
Ultrasonography measurement on vastus lateralis and the points of measurements on the skin **(A,B).** Sagittal plane panoramic ultrasound image of right leg’s m. quadriceps femoris—vastus lateralis of same individual; the red stars sign the measurement points **(C)**.

All imaging modalities were performed by an experienced radiologist specialist to avoid interobserver variability. Image acquisition was performed in a standardized patient position in relaxed and in maximal isometric contraction phase of the quadriceps femoris vastus lateralis muscle at the pre-determined angular position. To standardize the ultrasound measurements of cross-sectional diameters, three standardized points were defined: the one-third, one-half, and two-thirds points of the length of vastus lateralis (Figure 2).

For B-mode images we determined the anatomical (dA) and physiological (dF) cross-sectional diameter of the target muscle (quadriceps vastus lateralis) at each point at rest and in the maximal isometric contraction phase. Target muscle thickness measurements were performed in B-mode scan at the three standardized points mentioned above (Figure 2). The anatomical and physiological diameter values were given in mm. To avoid compression errors, the US probe was placed above a given site without any pressure and with the use of a thick layer of US gel between the probe and the skin.

The same US equipment with a L9 9 MHz probe was used to quantify tissue stiffness by measuring the speed of shear waves in m. quadriceps femoris—vastus lateralis. The dA, dF variables were also measured with the same US probe. As before, compression artefacts were avoided with the use of a thick layer of US gel between the probe and the skin. The target muscle of the athlete was visualized through the use of US. Elasticity boxes were established in the center of the field of view. Shear wave elastography images were saved after a few seconds of immobilization.

For measurement, circular region of interest (ROI) was generated inside of the elasticity box, and the stiffness was calculated in kPa. Elastography measurements were repeated at twelve different points in the vastus lateralis. In order to reduce the variability of the repeated measurements, an inter quartile range-based outlier detection was performed in each case (14,25).

### Statistical Methods

The basic data was characterized by means and standard deviations. To determine normality, because of the limited sample size and for the purpose of selecting the adequate statistical procedure, Shapiro Wilk’s W test was applied. Differences between the groups were compared with an independent *t*-test when the data was normally distributed, and if this was not the case, a Mann–Whitney *U* test was applied. If more than two samples were compared, to minimize the possibility of Type 1 error, we have calculated one way ANOVA and subsequently Tukey HSD post hoc test for unequal sample sizes. A Pearson’s correlation analysis was performed. Data analysis was performed on Statistica 12 program (Statsoft Statistica, TIBCO Software Inc., United States); the significance level was determined to be *p* < 0.05.

## Results

### Comparison of Samples

The comparison of athletes and non-athletes regarding the body composition data resulted in significantly larger values for the non-athletes in BF% (43.2%, *p* < 0.05) and significantly larger values for the athletes in BM% (20.6%, *p* < 0.05). In the other body composition variables there was no significant difference between the athletes and non-athletes. For the body composition parameters there was no difference between the H and T groups.

When we compared the F and S samples for the body composition data we have found significant differences in the following variables: in BH F was 13% greater than S (*p* < 0.05); in BW F was 66% greater than S (*p* < 0.05); in BMI F was 27% greater than S (*p* < 0.05). For the following body composition variables, there was no significant difference: BF, VF, BM (Table 1).

**TABLE 1:** Body composition data (mean ± SD) for the samples.

Sample	BH (cm)*	BW (kg)*	BF (%)	VF (%)	BM %	BMI*
F	188.7 ± 6.2	90.3 ± 16.4	17.6 ± 6.8	6.5 ± 3.7	40.4 ± 4.2	25.3 ± 4.1
S	166.2 ± 6.9	54.6 ± 12	19.1 ± 5.2	4 ± 2.8	36.1 ± 4.5	19.7 ± 2.5
H	174.5 ± 11.6	70 ± 15,9	25.3 ± 5.5	5.4 ± 2.6	33.7 ± 4.9	22.7 ± 2.5
T	173.5 ± 10.8	69.0 ± 18.9	27.6 ± 5.0	5.3 ± 2.6	30.9 ± 3.9	22.5 ± 3.2

*Indicates significant difference between F and S sample; + indicates significant difference between athlete (F + S) and non-athlete (H + T), p < 0.05.

F, fast muscle fiber dominance athlete group; S, slow muscle fiber dominance athlete group; H, healthy non-athlete group; T, group of cancer patients.

H, body height; BW, body weight; BF, body fat percentage; VF, visceral fat percentage; BM, body muscle percentage; BMI, body mass index.

The ultrasound data for the athletes and non-athletes showed significantly greater values for the athletes in isometric max and also when the muscle was in a resting state: dA[mm]I, dA[mm]II, dF[mm]I, dF[mm]II (*p* < 0.05).

The statistical calculations for *E* (kPa) for the athletes and non-athletes resulted significantly greater values (31.1%, *p* < 0.05) when isometric max was applied for athletes than for non-athletes. In the resting state there was no significant difference between athletes and non-athletes in terms of *E* (kPa) values.

The H and T sample comparison indicated significantly greater *E* (kPa) variable for H than for T (37.9%, *p* < 0.05). For the other ultrasound variables there was no significant difference between the H and T samples.

When we evaluated the ultrasound data for the F and S samples, when isometric max was applied showed, significant differences were found in the following variable: in *E* (kPa) F was 57% greater than S (*p* < 0.05). For the following ultrasound variables, there was no significant differences in terms of the isometric max contraction: dA, dF. The comparison of the ultrasound data for the F and S samples when the muscle was in a resting state indicated no significant differences in the E, dA, dF variables (Tables 2 and 3).

**TABLE 2:** Ultrasound data in isometric max contraction of the muscle for the samples.

Subjects	E (kPa)*	dA (mm) I.	dF (mm) I.	dA (mm) II.	dF (mm) II.	dA (mm) III.	dF (mm) III.
F	44.4 ± 6.2	27.2 ± 0.7	29.7 ± 4.0	27.3 ± 0.8	29.3 ± 0.8	19.5 ± 0.6	20.5 ± 0.7
S	28.2 ± 3.5	17.0 ± 0.7	18.3 ± 4.4	18.9+0.4	21.2 ± 0.4	18.3 ± 0.2	21.9 ± 0.4
H	28.6 ± 25.8	16 ± 15.7	18.8 ± 2.3	21.3 ± 4.2	14.9 ± 1.9	19.4 ± 3.0	19.4 ± 3.0
T	20.7 ± 24.6	15.3 ± 15.7	18.1 ± 18.5	18.6 ± 19.5	14.9 ± 15	20.0 ± 19.2	20.0 ± 19.2

*Indicates significant difference between F and S sample; + indicates significant difference between athlete (F + S) and non-athlete (H + T), p < 0.05.

F, fast muscle fiber dominance athlete group; S, slow muscle fiber dominance athlete group; H, healthy non-athlete group; T, group of cancer patients.

E, mean elasticity values; dA, anatomical cross–sectional diameter; dF, physiological cross-sectional diameter.

**TABLE 3:** Ultrasound data during resting phase of the muscle for the samples.

Subjects	E (kPa) I.	dA (mm) I.	dF (mm) I.	dA (mm) II.	dF (mm) II.	dA (mm) III.	dF (mm) III.
F	21.3 ± 3.5	25.6 ± 0.7	27.1 ± 4.1	26.7 ± 0.6	28.6 ± 0.7	19.6 ± 0.7	20.5 ± 0.7
S	17.8 ± 1.8	20.2 ± 0.5	22 ± 0.5	20.2 ± 0.3	21.7 ± 0.3	19.0 ± 0.3	20.7 ± 0.3
H	17.3 ± 4.6	19.8 ± 4.3	21.4 ± 4.5	20.3 ± 4.4	23 ± 4.3	16.6 ± 3.2	18.8 ± 3.8
T	17.3 ± 1.9	18.7 ± 5.7	20 ± 5.6	17.4 ± 1.9	20.0 ± 3.3	16 ± 2.8	17.5 ± 3.1

*Indicates significant difference between F and S sample; + indicates significant difference between athlete (F + S) and non-athlete (H + T), p < 0.05.

F, fast muscle fiber dominance athlete group; S, slow muscle fiber dominance athlete group; H, healthy non-athlete group; T, group of cancer patients.

E, mean elasticity values; dA, anatomical cross–sectional diameter; dF, physiological cross-sectional diameter.

When we compared the data for the athletes and non-athletes in terms of the dynamometric data indicated significantly greater values for the athletes in Mmax (112.2% *p* < 0.05). RTD was not measured for the non-athlete sample for safety reasons. The comparison of H and T samples resulted in significantly greater Mmax values for H than for T (136%, *p* < 0.05).

The comparison of the F and S samples for the dynamometric data resulted in significant differences in the following variables: in the quadriceps isometric Mmax, F was 71% greater than S; in RTDk F was 137% greater than S (*p* < 0.05). In the following dynamometric variables there was no significant difference: RTDr; torque/weight (M/weight) (Table 4).

**TABLE 4:** Dynamometric data (mean ± SD) for the F and S athletes’ samples.

Sample	Mmax (Nm)*	RTDk (Nm/s)*	RTDr (Nm/s)
F	325.8 ± 37.5	8.2 ± 1.3	1.6 ± 0.5
S	190.1 ± 28.8	5.9 ± 0.2	1.16 ± 0.1

*Significant difference between F and S sample (p<0.05).

F, fast muscle fiber dominance athlete group; S, slow muscle fiber dominance athlete group.

Mmax, maximal isometric torque; RTDk, rate of tension development at contraction; RTDr, rate of tension development at release.

### Correlation Between Variables

To execute the correlational calculations, a correlation matrix was constructed, including all the data for the sample. For this paragraph only the correlations which were important for the purpose of the study were highlighted.

### Correlations Between Body Composition and Dynamometric Variables

From the measured data correlation could be found between the following variables for the non-athlete (H + T) sample (*p* < 0.05): BH-Mmax (*r* = 0.64); BW-Mmax (*r* = 0.62); BM%-Mmax (*r* = 0.68). The calculations indicated a correlation between the following variables for the athlete (F + S) sample (*p* < 0.05): BH-Mmax (*r* = 0.77); H-RTDk (*r* = 0.77); BW-Mmax (*r* = 0.76); BF%-RTDk (*r* = 0.83); BF%-Mmax/kg (*r* = −0.83).

### Correlations Between Body Composition and Ultrasound Variables

**FIGURE 3:**
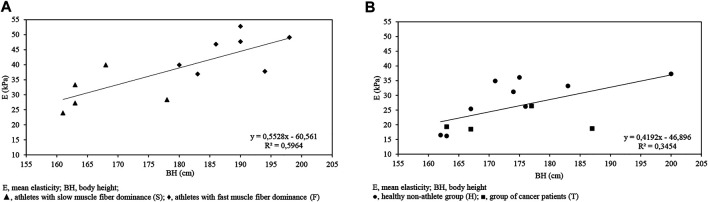
BH-E (kPa) graph indicating a correlation between variables for the athlete (F + S) sample (*r* = 0.76, *p* < 0.05) **(A)** and for the non-athlete (H + T) sample (*r* = 0.58, *p* < 0.05) **(B)**.

**FIGURE 4:**
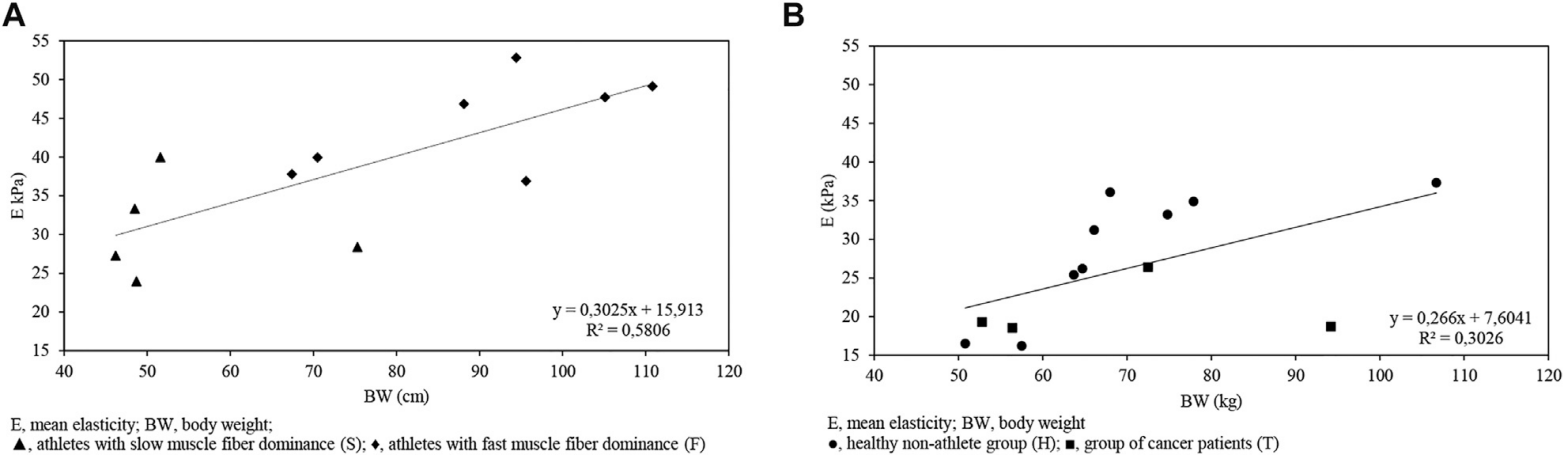
BW-E (kPa) graph indicating a correlation between variables for the athlete (F + S) sample (*r* = 0.76, *p* < 0.05) **(A)** and for the non-athlete (H + T) sample (*r* = 0.55, *p* = 0.058) **(B)**.

The calculations indicated a correlation between the following variables for isometric max contraction ultrasound data and body composition data for the athlete (F + S) sample (*p* < 0.05): BW-dAI. (*r* = 0.89); BW-dFI. (*r* = 0.87); BW-dAII. (*r* = 0.72); BW-dFII. (*r* = 0.72); BMI-dAI. (*r* = 0.93); BMI-dFI. (*r* = 0.92); BMI-dAII. (*r* = 0.78); BMI-dFII. (*r* = 0.8). There was a significant correlation between BH-E (*r* = 0.76) and BW-E (*r* = 0.77) (Figures 3 and 4).

After calculating the correlation between the following variables for isometric max contraction ultrasound data and body composition data for the non-athlete (H + T) sample (*p* < 0.05) we have determined the following r value: BH-dFIII. (*r* = 0.63); BH-E (*r* = 0.58) (Figures 3 and 4).

Correlation was found between the following variables for resting muscle state and ultrasound data for the athlete (F + S) sample (*p* < 0.05): BF-dFII. (*r* = 0.67); BF-dAIII. (*r* = 0.76); BF-dFIII. (0.74); VF-dAII. (*r* = 0.71); VF-dFII. (*r* = 0.68); VF-dAIII. (*r* = 0.73); VF-dFIII. (*r* = 0.68).

The calculations indicated a correlation between the following variables for resting muscle state and ultrasound data for the non-athlete (H + T) sample (*p* < 0.05): BH-E (kPa) (*r* = 0.64).

### Correlations Between Ultrasound and Dynamometric Variables

**FIGURE 5:**
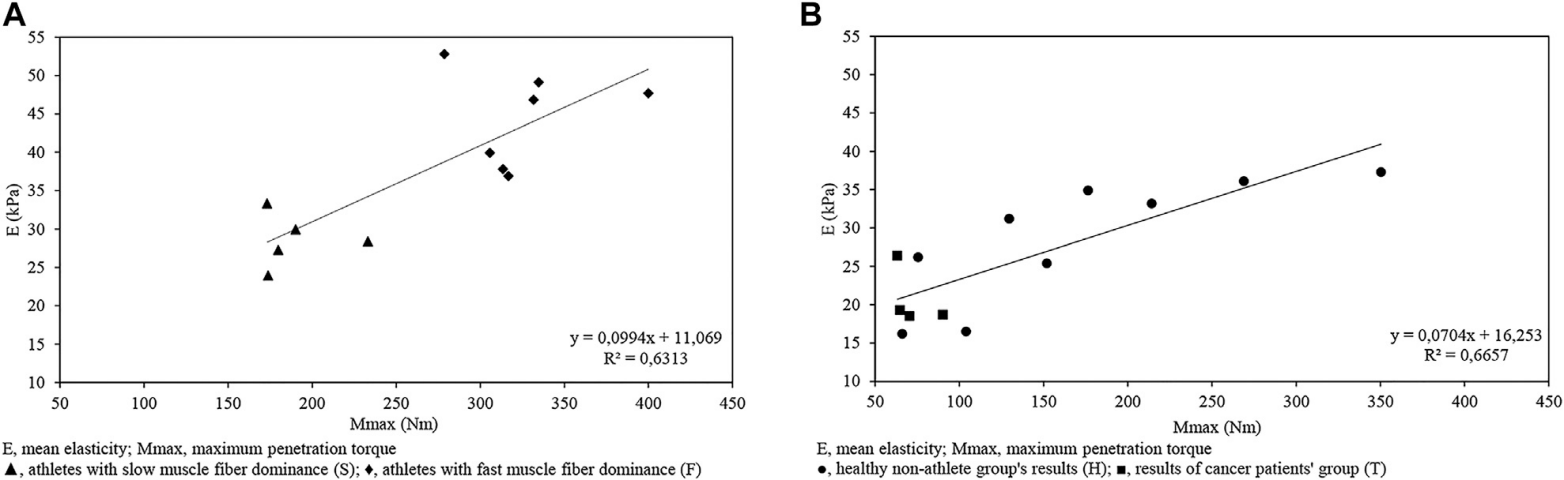
Mmax-E (kPa) graph indicating a significant correlation between variables for the athlete (F + S) sample (*r* = 0.79; *p* < 0.05) **(A)**; and for the non-athlete (H + T) sample (*r* = 0.816, *p* < 0.05) **(B)** measured in isometric max contraction.

As in Figure 5A, the calculations indicated a correlation between the following variables for isometric max contraction and ultrasound data for the athlete (F + S) sample (*r* = 0.79, *p* < 0.05).

The difference between the athlete participants with explosive muscle fiber dominance and endurance athletes in terms of *E* (kPa) was significant (*p* < 0.05).

The correlation calculation between the following variables for isometric max contraction and ultrasound data for the non-athlete (H + T) sample resulted in significant relation (*r* = 0.816, *p* < 0.05); (Figure 5B).

**FIGURE 6:**
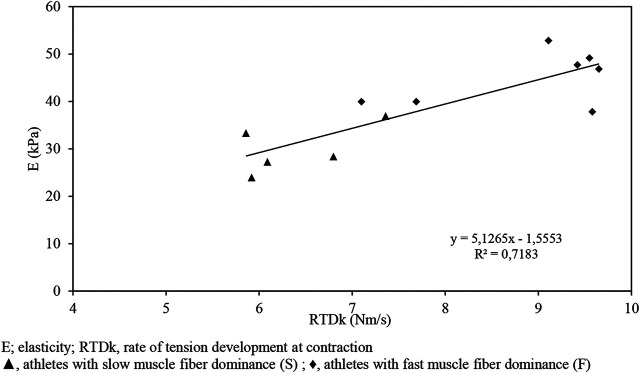
RTDk-E graph indicating a correlation between variables for the athlete (F + S) sample (*r* = 0.84) measured in isometric max contraction.

The strongest correlation characterizes the variables for RTDk and ultrasound data (*r* = 0.84, *p* < 0.05) presented in Figure 6.

## Discussion

As the determination of the dynamic parameters of a human muscle can be extremely beneficial in the treatment and rehabilitation of subjects suffering from an illness which has a direct or indirect effect on individual muscle characteristics as well as in the training of elite athletes, our aim was to examine the possibilities of ultrasound detection in muscle dynamic characteristics measurements (26). The correlations between BH-Mmax and BW-Mmax for both the athletes and non-athletes was in accordance with previous studies (27), as for the athletes of similar body composition with greater body mass, the results showed greater muscle mass, which consequently resulted in greater Mmax. The investigation of the ultrasound data indicated a correlation between *E* (kPa) and Mmax, for both the athletes and non-athletes; for the non-athletes we calculated an even greater *r* value. For the athletes we measured a correlation between E and RTDk when maximal isometric contraction was applied. From this result we can conclude that if maximal isometric contraction can be achieved, then the data for E that can be measured through the application of ultrasound can be used to gather information about the dynamic properties of the human muscle. This technique can be beneficial when the measurement of muscle tension is not possible with the use of a dynamometer as a result of a joint injury (e.g., ACL injury), when a dynamometer is not available, and in cases where the knee cannot be bent; therefore, using a dynamometer is impossible, or data is needed about the contraction characteristics of one individual muscle, for example, in the monitoring of degradation in muscle tension due to cancer or treatment. We must also stress that as we investigated body composition, *E* (kPa), and Mmax values and looked for correlations between variables, we concluded that in our study the best indicator of the maximal isometric force capacity of the muscle was the similar ultrasound *E* (kPa) values for athletes and non-athletes. It is worth mentioning that in the case of tumors, muscle volume loss is a direct consequence of the disease and treatment; in older age muscle mass loss, sarcopenia is a natural process whereas after a physical injury, muscle mass and loss occur as a result of immobilization (28–30). In addition to this, the study of muscle dystrophies (Dystrophia myotonica I-II, Facioscapulohumeral muscular dystrophy, Limb-girdle muscular dystrophy, Duchenne/Becker, Mitochondrialis, Pompe) might be another area of application for our method as hereditary muscle disorder, for example, the histology and histopathology features of dystrophia myotonica (I.II.), also known as Steinert’s disease (Myotonic dystrophy type 1 and type 2), are very similar and exhibit complex muscle structure abnormalities (31–40). The procedure can also be used with individuals suffering from paraplegia, where maximal muscle tension can be evoked with external electrical stimulus.

Focusing on the results of cancer patients, the results indicate significantly smaller Mmax values for these patients (T) than for the healthy non-athletes (H), although in terms of body composition parameters, there was no difference between the healthy and patient groups. This result indicates that the force-generation properties are significantly lower for these cancer patients. Presumably, with a decrease in muscle force, immobilization and hospitalization will follow. Therefore, to preserve a cancer patient’s mobility it is highly beneficial for the individual as well as the healthcare system, as our results demonstrate, that this technology can support this goal.

**FIGURE 7:**
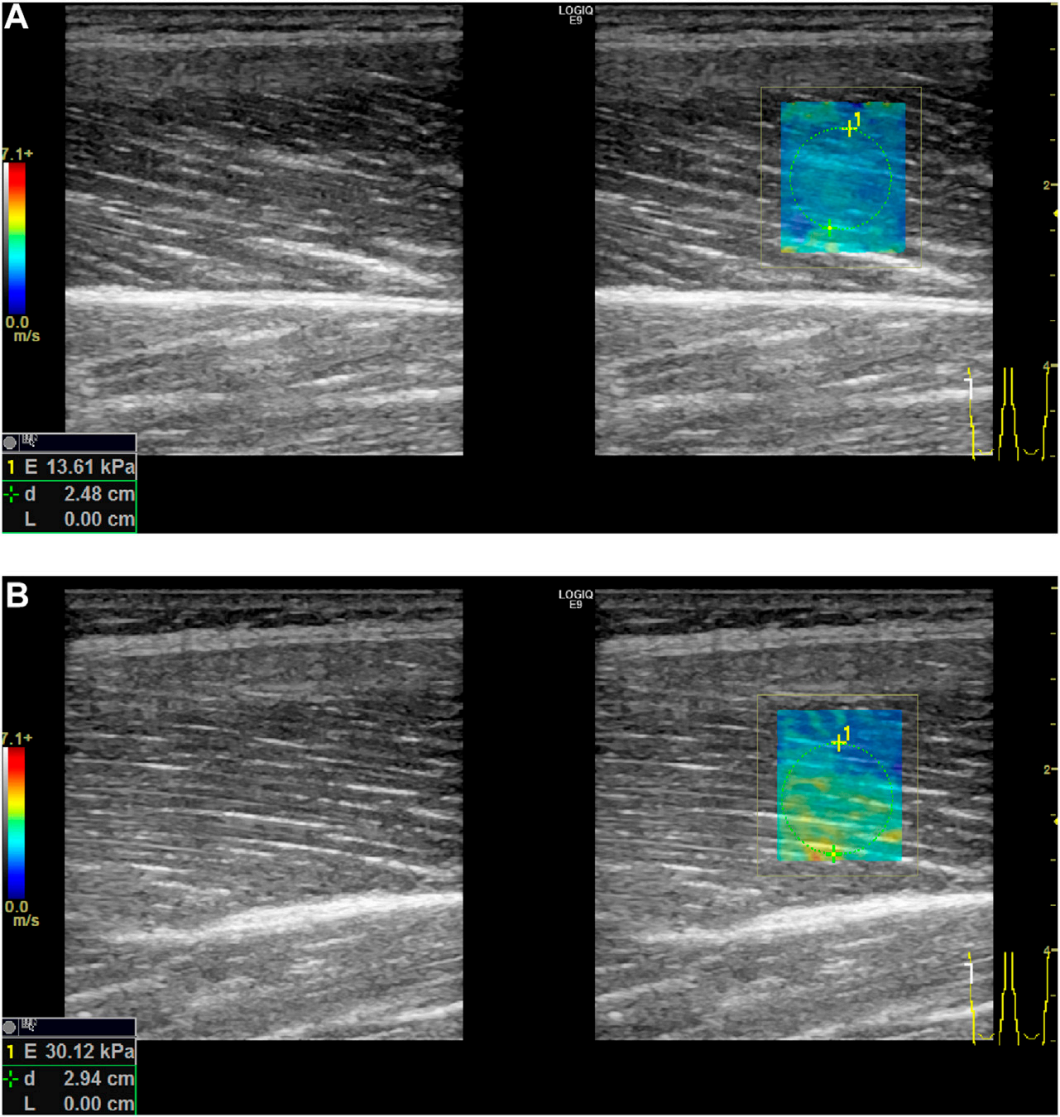
Differences in mean elasticity values between relaxation **(A)** and contraction **(B)** phase in shear wave examination of right leg’s m. quadriceps femoris—vastus lateralis (*E*, mean elasticity value; *d*, depth).

As the muscle maximal isometric force is highly dependent upon muscle fiber type, we investigated two groups, S and F. We only investigated these two groups in this stage of the study because concerning S and F samples the specified sports background allows us to approximate muscle type dominance for the two groups. Our results indicated that for the F sample significantly greater results could be seen in terms of height, BW, and BMI, and, consequently, Mmax. This result is in accordance with the conclusions of previous studies (41). The significant difference in RTDk (explosive force) for the S and F samples thus indicates a difference in muscle fiber type dominance for the samples and, therefore, validates our differentiation between the samples. When the ultrasound data was compared for the F and S samples, a significant difference was found regarding E when the muscle performed maximal isometric voluntary contractions. Based on our results concerning the differences in E values for the explosive and endurance athletes, we can conclude that the method of using shear wave ultrasound technology can be useful in the determination of muscle fiber dominance. As muscle biopsy is not an ethically approved method, especially for children, ultrasound technology can be used to determine the appropriate sport for young individual athletes (Figure 7).

The sensitivity of ultrasound results in terms of muscle fiber ratio can also be beneficial concerning the rehabilitation of cancer patients. According to recent studies, in the case of cancer patients with a decrease in muscle mass, a simultaneous shift into fast-twitch muscle fibers from slow-twitch fibers occurs; with ultrasound elastography this phenomenon can be tracked, and based on the measured data, the rehabilitation protocol of the individual patient can be altered accordingly (30).

It should be stressed that this is only a preliminary study, through which we would like to highlight the possibilities of ultrasound elastography measurement in the determination of muscle contraction properties and, at the same time, the fact that many limitations occur concerning the procedure. We are aware that the sample size is limited, especially concerning tumor patients, but with the current state of coronavirus lockdown restrictions we were fortunate that we were able to integrate these patients into the study. We also know that the determination of S and F samples can be seen as speculative, but as the execution of muscle biopsies are not acceptable for ethical reasons, we were only able to rely on indirect data concerning the muscle type ratio previously determined for top-level athletes in specific sports fields.

Our aim was to investigate the possible applications of quantitative shear wave elastography in the determination of mechanical properties, such as maximal tension of the skeletal muscles. We can conclude that if a maximal voluntary contraction can be performed and, simultaneously, E in the individual muscle can be measured through the use of ultrasound, this would be a useful tool in estimating the tension that can be generated by the individual muscle at maximal isometric contraction. This non-invasive, non-toxic measurement could be frequently repeated, providing important information on muscle status for the definition and evaluation of muscle tension degradation in cancer treatment and rehabilitation, the determination of sarcopenia, and, also, to determine the training load of an elite athlete and even for the future’s athletes at youth age by the time of choosing a sport.

The physical exercise and skeletal muscle status have paramount importance in prevention, in treatment support and in rehabilitation of a large number of different diseases including musculoskeletal and neurodegenerative disorders, critically ill patients with different chronic metabolic diseases and various types of cancer. Therefore, the definition of appropriate, personalized training, objective follow up on its effectivity using non-invasive method is of high interest for different medical conditions. The relatively simple SWE measurement could be applied to evaluate the skeletal muscle status, to monitor the contractile property changes over time (18,42) during the development, treatment and rehabilitation of these diseases. High shear modulus is associated with muscle stiffness in cerebral palsy or in late Duchenne’s myopathy and Parkinson’s disease (43,44), while low shear modulus is associated with atrophy in a GNE chronic myopathy (45). Moreover, shear modulus is associated with muscle fibrosis after radiotherapy. SWE could be performed easily at the bed side of critically ill population at high risk of muscle edema (46). In the recent years special interest was focused on physical training for cancer patients at the different stage of the malignant disease development. A clear evidence supports an association between regular physical activity (PA) and decreased risk for cancer and cancer mortality (47). After diagnosis of a malignant disease the physical exercise contributes to improved health and functional outcomes (48,49). Qualitative and quantitative skeletal muscle assessment demonstrated independent associations with fitness and clinical outcomes among cancer survivors (50,51). For example, physical prehabilitation programs could prepare patients for surgery by improving their functional status with the aim of reducing postoperative complications (52,53). The number of cancer survivors with less complications is continuously increasing due to improved screening, diagnoses, imaging, endoscopic-, histopathologic and molecular examination methods, and interdisciplinary defined treatments using advanced technology in local therapeutic modalities and effective novel approaches such as molecular targeted systemic therapy and immunotherapy and their combination. Cancer and sport medicine research proved the benefits of regular physical activity including aerobic, resistance training and muscle stretching (54–57). SWE is an important tool of quantitative monitoring of muscle status in the growing scientific interest on exercise intervention integrated in the cancer management.

The definition of fiber type could be important information in defining the appropriate individual training program or physiotherapy for disabled patients. Physical exercises —particularly those that incorporate resistance training—are effective in reversing, preventing, or mitigating skeletal muscle loss. The personalized training programs adapted to the patients’ muscle constitution may lead to better compliance and higher probability to improve physical functioning and body composition and to maintain or increases skeletal muscle mass and strength.

Predicting the fiber ratio in case of healthy young age groups is a forward-looking method in choosing the right sport because the nature of the sport presupposes the existence of different basic abilities.

In our study the sample size was restricted due to the complexity of the data collection and current coronavirus restrictions; we think that future investigations with greater sample sizes and different sample properties, for example, including a higher number of patients in the sample, could deepen our knowledge and, consequently, widen the possibilities for the usage of ultrasound techniques, both in the medical field and in the sport sciences.

## Data Availability

The raw data supporting the conclusions of this article will be made available by the authors, without undue reservation.

## Abbreviations

ACL, Anterior cruciate ligament; BF(%), Body fat percentage; BH (cm), Body height; BM(%), Body muscle percentage; BMI, Body mass index; BW(Kg), Body weight; *E* (kPa), Mean elasticity values in kPa (kilopascal) unit, stiffness; DA, Anatomical cross-sectional diameter; DF, Physiological cross-sectional diameter; F, Fast muscle fiber dominance group, group of strength athletes; H, Group of healthy, non-athlete subjects; Mmax (Nm), Maximal isometric torque; M/weight (Nm/kg), Torque/weight; ROI, Region of interest; RTDk (Nm/s), Rate of tension development at contraction; RTDr (Nm/s), Rate of tension development at release; S, Slow muscle fiber dominance group, group of endurance athletes; SA, Statistical age; SWE, Shear wave elastography; T, Group of cancer patients; VF (%), Visceral fat percentage; US, Ultrasound.

## References

[1] Valente KP Almeida BL Lazzarini TR Souza VFd. Ribeiro Td. SC Guedes de Moraes RA Association of Adductor Pollicis Muscle Thickness and Handgrip Strength with Nutritional Status in Cancer Patients. PLoS One (2019) 14:e0220334. 10.1371/journal.pone.0220334 31374093 PMC6677294

[2] Barata AT Santos C Cravo M Vinhas Md. C Morais C Carolino E Handgrip Dynamometry and Patient-Generated Subjective Global Assessment in Patients with Nonresectable Lung Cancer. Nutr Cancer (2017) 69:1154–8. 10.1080/01635581.2017.1250923 27918868

[3] Bishop DJ Girard O. Determinants of Team-Sport Performance: Implications for Altitude Training by Team-Sport Athletes. Br J Sports Med (2013) 47(1):i17–i21. 10.1136/bjsports-2013-092950 24282200 PMC3903139

[4] Abbott A Button C Pepping GJ Collins D. Unnatural Selection: Talent Identification and Development in Sport. Nonlinear Dyn Psychol Life Sci (2005) 9(1):61–88. 10.4324/9781003049111-4 15629068

[5] Pukkala E Kaprio J Koskenvuo M Kujala U Sarna S. Cancer Incidence Among Finnish World Class Male Athletes. Int J Sports Med (2000) 21(3):216–20. 10.1055/s-2000-8878 10834356

[6] Robsahm TE Hestvik UE Veierød MB Fagerlie A Nystad W Engebretsen L Cancer Risk in Norwegian World Class Athletes. Cancer Causes Control (2010) 21:1711–9. 10.1007/s10552-010-9600-z 20544266

[7] Wilson A Lichtwark G. The Anatomical Arrangement of Muscle and Tendon Enhances Limb Versatility and Locomotor Performance. Phil Trans R Soc B (2011) 366:15701540–1553. 10.1098/rstb.2010.0361 PMC3130454 21502125

[8] Bohannon RW. Considerations and Practical Options for Measuring Muscle Strength: A Narrative Review. Biomed Res Int (2019) 2019:1–10. 10.1155/2019/8194537 PMC6354207 30792998

[9] Knapik JJ Ramos MU. Isokinetic and Isometric Torque Relationships in the Human Body. Arch Phys Med Rehabil (1980) 61(2):64–7. 7369840

[10] Baltzopoulos V Brodie DA. Isokinetic Dynamometry. Sports Med (1989) 8(2):101–16. 10.2165/00007256-198908020-00003 2675256

[11] Thistle HG Hislop HJ Moffroid M Lowman EW. Isokinetic Contraction: A New Concept of Resistive Exercise. Acta Physiol Scand Suppl (1967) 48(6):279–82. 6026595

[12] Aagaard P Simonsen EB Magnusson SP Larsson B Dyhre-Poulsen P. A New Concept for Isokinetic Hamstring: Quadriceps Muscle Strength Ratio. Am J Sports Med (1998) 26(2):231–7. 10.1177/03635465980260021201 9548116

[13] Duarte JP Valente-Dos-Santos J Coelho-E-Silva MJ Couto P Costa D Martinho D Reproducibility of Isokinetic Strength Assessment of Knee Muscle Actions in Adult Athletes: Torques and Antagonist-Agonist Ratios Derived at the Same Angle Position. PLoS One (2018) 13:e0202261. 10.1371/journal.pone.0202261 30110385 PMC6093703

[14] Hug F Tucker K Gennisson J-L Tanter M Nordez A. Elastography for Muscle Biomechanics. Exerc Sport Sci Rev (2015) 43(3):125–33. 10.1249/JES.0000000000000049 25906424

[15] Yoshitake Y Takai Y Kanehisa H Shinohara M. Muscle Shear Modulus Measured with Ultrasound Shear-Wave Elastography across a Wide Range of Contraction Intensity. Muscle Nerve (2014) 50:103–13. 10.1002/mus.24104 24155045

[16] Roberts D Ageberg E Andersson G Fride T. Clinical Measurements of Proprioception, Muscle Strength and Laxity in Relation to Function in the ACL-Injured Knee. Knee Surg Sports Traumatol Arthrosc (2007) 15:19–6. 10.1007/s00167-006-0128-4 16791634

[17] Taljanovic MS Gimber LH Becker GW Latt LD Klauser AS Melville DM Shear-Wave Elastography: Basic Physics and Musculoskeletal Applications. Radiographics (2017) 37(3):855–70. 10.1148/rg.2017160116 28493799 PMC5452887

[18] Creze M Nordez A Soubeyrand M Rocher L Maître X Bellin M-F. Shear Wave Sonoelastography of Skeletal Muscle: Basic Principles, Biomechanical Concepts, Clinical Applications, and Future Perspectives. Skeletal Radiol (2018) 47(4):457–71. 10.1007/s00256-017-2843-y 29224123

[19] Brandenburg JE Eby SF Song P Zhao H Brault JS Chen S Ultrasound Elastography: The New Frontier in Direct Measurement of Muscle Stiffness. Arch Phys Med Rehabil (2014) 95(11):2207–19. 10.1016/j.apmr.2014.07.007 25064780 PMC4254343

[20] Andresen JL Schjerling P Saltin B. Muscles, Genes and Athletic Performance. Sci Am (2000) 283(3):48–55. 10.1038/scientificamerican0900-48 10976466

[21] Methenitis S Spengos K Zaras N Stasinaki A-N Papadimas G Karampatsos G Fiber Type Composition and Rate of Force Development in Endurance- and Resistance-Trained Individuals. J Strength Cond Res (2019) 33(9):2388–97. 10.1519/JSC.0000000000002150 28737590

[22] Methenitis S Terzis G Zaras N Stasinaki A-N Karandreas N. Intramuscular Fiber Conduction Velocity, Isometric Force and Explosive Performance. J Hum Kinet (2016) 51:93–101. 10.1515/hukin-2015-0174 28149372 PMC5260554

[23] Methenitis S Karandreas N Spengos K Zaras N Stasinaki A-N Terzis G. Muscle Fiber Conduction Velocity, Muscle Fiber Composition, and Power Performance. Med Sci Sports Exerc (2016) 48(9):1761–71. 10.1249/MSS.0000000000000954 27128672

[24] Sadoyama T Masuda T Miyata H Katsuta S. Fibre Conduction Velocity and Fibre Composition in Human Vastus Lateralis. Europ J Appl Physiol (1988) 57(6):767–71. 10.1007/BF01076001 3416864

[25] Taljanovic MS Gimber LH Becker GW Latt LD Klauser AS Melville DM Shear-wave Elastography: Basic Physics and Musculoskeletal Applications. Radiographics (2017) 37(3):855–70. 10.1148/rg.2017160116 28493799 PMC5452887

[26] Newman AB Haggerty CL Goodpaster B Harris T Kritchevsky S Nevitt M Strength and Muscle Quality in a Well-Functioning Cohort of Older Adults: the Health, Aging and Body Composition Study. J Am Geriatr Soc (2003) 51(3):323–30. 10.1046/j.1532-5415.2003.51105.x 12588575

[27] Hasan NAKAK Kamal HM Hussein ZA. Relation between Body Mass index Percentile and Muscle Strength and Endurance. Egypt J Med Hum Genet (2016) 17(4):367–72. 10.1016/j.ejmhg.2016.01.002

[28] Jackson W Alexander N Schipper M Fig L Feng F Jolly S. Characterization of Changes in Total Body Composition for Patients with Head and Neck Cancer Undergoing Chemoradiotherapy Using Dual-Energy X-ray Absorptiometry. Head Neck (2013) 36:a1356. 10.1002/hed.23461 23970480

[29] Antoun S Birdsell L Sawyer MB Venner P Escudier B Baracos VE. Association of Skeletal Muscle Wasting with Treatment with Sorafenib in Patients with Advanced Renal Cell Carcinoma: Results from a Placebo-Controlled Study. Jco (2010) 28:61054–1060. 10.1200/JCO.2009.24.9730 20085939

[30] Toth MJ Callahan DM Miller MS Tourville TW Hackett SB Couch ME Skeletal Muscle Fiber Size and Fiber Type Distribution in Human Cancer: Effects of Weight Loss and Relationship to Physical Function. Clin Nutr (2016) 35:61359–1365. 10.1016/j.clnu.2016.02.016 PMC6411286 27010836

[31] Mankodi A Logigian E Callahan L McClainC White R Henderson D Myotonic Dystrophy in Transgenic Mice Expressing an Expanded CUG Repeat. Science (2000) 289:1769–72. 10.1126/science.289.5485.1769 10976074

[32] Lukáš Z Kroupová I Bednařík J Falk M Fajkusová L Sedláčková J Muscular Biopsy in Myotonic Dystrophy in the Era of Molecular Genetics. Ceska a Slovanska Neurologia a Neurochirurgie (2007) 70:103–401. 10.5772/30209

[33] Taneja KL. Localization of Trinucleotide Repeat Sequences in Myotonic Dystrophy Cells Using a Single Fluorochrome-Labeled PNA Probe. Biotechniques (1998) 24(3):472–6. 10.2144/98243rr02 9526660

[34] Jiang H Mankodi A Swanson MS Moxley RT Thornton CA. Myotonic Dystrophy Type 1 Is Associated with Nuclear Foci of Mutant RNA, Sequestration of Muscleblind Proteins and Deregulated Alternative Splicing in Neurons. Hum Mol Genet (2004) 13:243079–3088. 10.1093/hmg/ddh327 15496431

[35] Thornton CA Griggs RC Moxley RT. Myotonic Dystrophy with No Trinucleotide Repeat Expansion. Ann Neurol (1994) 35(3):269–72. 10.1002/ana.410350305 8122879

[36] Liquori CL Ricker K Moseley ML Jacobsen JF Kress W Naylor SL Myotonic Dystrophy Type 2 Caused by a CCTG Expansion in Intron 1 of ZNF9. Science (2001) 293:5531864–7. 10.1126/science.1062125 11486088

[37] Day JW Ranum LPW. RNA Pathogenesis of the Myotonic Dystrophies. Neuromuscul Disord (2005) 15:15–6. 10.1016/j.nmd.2004.09.012 15639115

[38] Falk M Vojtíšková M Lukáš Z Kroupová I Froster U. Simple Procedure for Automatic Detection of Unstable Alleles in the Myotonic Dystrophy and Huntington's Disease Loci. Genet Test (2006) 10:85–97. 10.1089/gte.2006.10.85 16792511

[39] Vihola A Bassez G Meola G Zhang S Haapasalo H Paetau A Histopathological Differences of Myotonic Dystrophy Type 1 (DM1) and PROMM/DM2. Neurology (2003) 60:1854–7. 10.1212/01.wnl.0000065898.61358.09 12796551

[40] Day JW Ricker K Jacobsen JF Rasmussen LJ Dick KA Kress W Myotonic Dystrophy Type 2. Neurology (2003) 60:4657–664. 10.1212/01.wnl.0000054481.84978.f9 12601109

[41] Hayashida I Tanimoto Y Takahashi Y Kusabiraki T Tamaki J. Correlation between Muscle Strength and Muscle Mass, and Their Association with Walking Speed, in Community-Dwelling Elderly Japanese Individuals. PLoS One (2014) 9:e111810. 10.1371/journal.pone.0111810 25365291 PMC4218822

[42] Mourtzakis M Parry S Connolly B Puthucheary Z. Skeletal Muscle Ultrasound in Critical Care: a Tool in Need of Translation. Ann ATS (2017) 14:1495–503. 10.1513/AnnalsATS.201612-967PS PMC5718569 28820608

[43] Lacourpaille L Hug F Guével A Péréon Y Magot A Hogrel J-Y Non-invasive Assessment of Muscle Stiffness in Patients with Duchenne Muscular Dystrophy. Muscle Nerve (2015) 51(2):284–6. 10.1002/mus.24445 25187068

[44] Carpenter EL Lau HA Kolodny EH Adler RS. Skeletal Muscle in Healthy Subjects versus Those withGNE-Related Myopathy: Evaluation with Shear-Wave US-A Pilot Study. Radiology (2015) 277(2):546–54. 10.1148/radiol.2015142212 26035587

[45] Du L-j. He W Cheng L-g. Li S Pan Y-s. Gao J. Ultrasound Shear Wave Elastography in Assessment of Muscle Stiffness in Patients with Parkinson's Disease: a Primary Observation. Clin Imaging (2016) 40:61075–1080. 10.1016/j.clinimag.2016.05.008 27408992

[46] Flatres A Aarab Y Nougaret S Garnier F Larcher R Amalric M Real-time Shear Wave Ultrasound Elastography: a New Tool for the Evaluation of Diaphragm and Limb Muscle Stiffness in Critically Ill Patients. Crit Care (2020) 24:1. 10.1186/s13054-020-2745-6 32014005 PMC6998330

[47] Stout NL Baima J Swisher A Winters-Stone KM Welsh J. A Systematic Review of Exercise Systematic Reviews in the Cancer Literature. PM&R.(2005). 9. S347–S384. 10.1016/j.pmrj.2017.07.074 PMC5679711 28942909

[48] Moore SC Lee I-M Weiderpass E Campbell PT Sampson JN Kitahara CM Association of Leisure-Time Physical Activity with Risk of 26 Types of Cancer in 1.44 Million Adults. JAMA Intern Med (2016) 176(6):816–25. 10.1001/jamainternmed.2016.1548 27183032 PMC5812009

[49] Lee J Lin JB Wu MH Jan YT Chang CL Huang CY Muscle Radiodensity Loss during Cancer Therapy Is Predictive for Poor Survival in Advanced Endometrial Cancer. J Cachexia, Sarcopenia Muscle (2019) 10(4):814–26. 10.1002/jcsm.12440 31094101 PMC6711455

[50] Weinberg MS Shachar SS Muss HB Deal AM Popuri K Yu H Beyond Sarcopenia: Characterization and Integration of Skeletal Muscle Quantity and Radiodensity in a Curable Breast Cancer Population. Breast J (2018) 24(3):278–84. 10.1111/tbj.12952 29139618 PMC6414810

[51] Ruiz-Casado A Martín-Ruiz A Pérez LM Provencio M Fiuza-Luces C Lucia A. Exercise and the Hallmarks of Cancer. Trends Cancer (2017) 3:423–41. 10.1016/j.trecan.2017.04.007 28718417

[52] Piraux E Reychler G de Noordhout LM Forget P Deswysen Y Caty G. What Are the Impact and the Optimal Design of a Physical Prehabilitation Program in Patients with Esophagogastric Cancer Awaiting Surgery? A Systematic Review. BMC Sports Sci Med Rehabil (2021) 13:1. 10.1186/s13102-021-00260-w 33766107 PMC7993458

[53] Campbell KL Winters-Stone KM Wiskemann J May AM Schwartz KS Exercise Guidelines for Cancer Survivors: Consensus Statement from International Multidisciplinary Roundtable. Med Sci Sports Exerc (2019) 51:112375–2390. 10.1249/MSS.0000000000002116 PMC8576825 31626055

[54] Ungar N Tsiouris A Haussmann A Herbolsheimer F Wiskemann J Steindorf K To Rest or Not to Rest-Health Care Professionals' Attitude toward Recommending Physical Activity to Their Cancer Patients. Psycho‐Oncology (2019) 28:4784–791. 10.1002/pon.5020 30716190

[55] Rock CL Doyle C Demark-Wahnefried W Meyerhardt J Courneya KS Schwartz AL Nutrition and Physical Activity Guidelines for Cancer Survivors. CA: A Cancer J Clinicians (2012) 62(4):242–74. 10.3322/caac.21142 22539238

[56] Nakano J Hashizume K Fukushima T Ueno K Matsuura E IKio Y Effects of Aerobic and Resistance Exercises on Physical Symptoms in Cancer Patients: A Meta-Analysis. Integr Cancer Ther (2018) 17:41048–1058. 10.1177/1534735418807555 PMC6247562 30352523

[57] Lobelo F Rohm Young D Sallis R Garber MD Billinger SA Duperly J Routine Assessment and Promotion of Physical Activity in Healthcare Settings: A Scientific Statement from the American Heart Association. Circulation (2018) 137:18:e495–e522. 10.1161/CIR.0000000000000559 29618598

